# Puberty Induction in Adolescent Males: Current Practice

**DOI:** 10.7759/cureus.23864

**Published:** 2022-04-05

**Authors:** Mohammed S Alenazi, Ali M Alqahtani, Mohammad M Ahmad, Eyad M Almalki, Angham AlMutair, Mussa Almalki

**Affiliations:** 1 Diabetes and Endocrine Treatment Center, Prince Sultan Military Medical City, Riyadh, SAU; 2 Obesity, Endocrine, and Metabolism Center, King Fahad Medical City, Riyadh, SAU; 3 Medicine, Shaqra University, Shaqra, SAU; 4 Pediatrics, Division of Endocrinology, King Abdulaziz Medical City, King Abdullah Specialist Children's Hospital, Ministry of National Guard-Health Affairs, Riyadh, SAU; 5 Medicine, King Abdulaziz Medical City, King Saud Bin Abdulaziz University for Health Sciences, Ministry of National Guard-Health Affairs, Riyadh, SAU; 6 Medicine, King Fahad Medical City, King Saud bin Abdul Aziz University for Health Sciences, Riyadh, SAU

**Keywords:** and gnrh therapy., gonadotropins, testosterone, hypogonadism, pubertal delay, boys, adolescent

## Abstract

Puberty is a developmental stage characterized by the appearance of secondary sexual characteristics which leads to complete physical, psychosocial, and sexual maturation. The current practice of hormonal therapy to induce puberty in adolescent males is based on published consensus and expert opinion. Evidence-based guidelines on optimal timing and regimen in puberty induction in males are lacking, and this reflects some discrepancies in practice among endocrinologists. It is worth mentioning that the availability of various hormonal products in markets, their different routes of administration, and patients/parents’ preference also have an impact on clinical decisions. This review outlines the current clinical approach to delayed puberty in boys with an emphasis on puberty induction.

## Introduction and background

**Figure 1:**
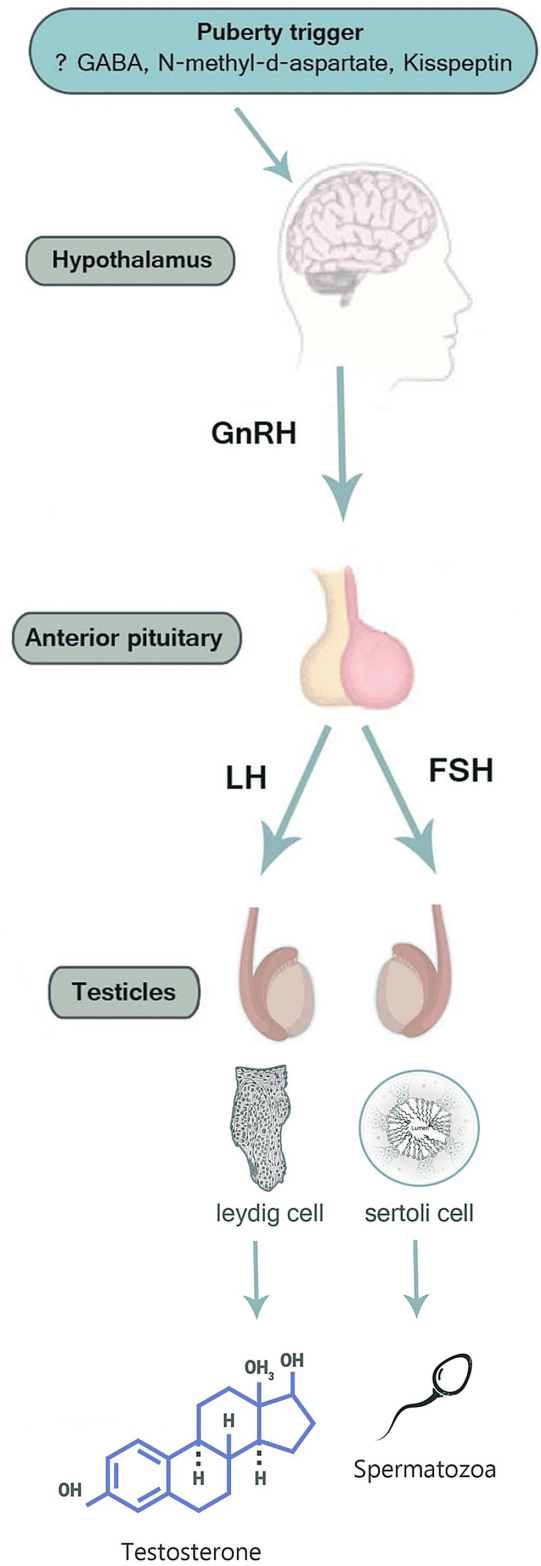
Normal puberty physiology.

Delayed puberty in males is defined as the absence of testicular growth at an age that is 2 to 2.5 SD later than the population means (traditionally, the age of 14 years). However the onset of puberty varies by country, race, and ethnicity [1], and it is delayed in around 2%-3% of boys [2]. Normal pubertal development is the result of the increasing release of gonadotropin-releasing hormone (GnRH) by the hypothalamus, which in turn stimulates the pituitary gland to release luteinizing hormone (LH) and follicle-stimulating hormone (FSH). Transient activation of the hypothalamus-pituitary-gonadal axis starts from intrauterine life to the first few months of life, a process that has been described as “mini-puberty.” Subsequently, the hypothalamus-pituitary-gonadal (HPG) axis is inactivated by gamma-aminobutyric acid (GABA) until the beginning of pubertal maturation [3]. The exact trigger that initiates pulsatile GnRH secretion is not fully known but is thought to be influenced by multiple factors including genetics, nutrition, neurotransmitters, and hormones. It has been demonstrated that the major neurotransmitter responsible for activating the GnRH pulse generator are glutamate, neuropeptide Y, endorphins, opioids, and melatonin [4]. Furthermore, kisspeptin and its receptor regulate GnRH secretion [4]. Inactivating mutations in the genes encoding the human kisspeptin receptor leads to failure of puberty progression [5]. The gonadotropins stimulate the development of gonads and result in synthesis as well as the release of sex steroids estrogens and androgens, and this process leads to the physical and hormonal changes of puberty: gonadarche indicates pubertal onset and it is provoked by the GnRH release in a pulsatile fashion, which activates the HPG axis. In males, LH stimulates the Leydig cells to produce testosterone and maintain spermatogenesis, while FSH stimulates the Sertoli cells and initiates spermatogenesis [6,7]. Adrenarche (i.e., androgen production by adrenal glands leading to the development of the pubic and axillary hairs, the sebaceous and the apocrine glands) is a separate but usually parallel process and does not in itself indicate genuine puberty [7]. Premature adrenarche is the presence of secondary sexual hairs in boys younger than nine years old [8]. The normal physiology of puberty is illustrated in Figure 1.

## Review

When to suspect delayed puberty in boys? 

Puberty is considered delayed when there are no signs of testicular enlargement by 14 years of age. The earliest indicator of genuine puberty in boys is a testicular enlargement of at least 4 mL in volume or 2.5 cm in length, which occurs at an average age of 11.6 years (range: 9.5 to 14 years) [9,10]. Any disruption of the normal physiology as described above may result in delayed puberty and under-virilization. And can also result in malformation of the external genitalia if the disruption occurs early in intrauterine life. In boys, once puberty has begun, a period of 3.2±1.8 years is necessary to achieve an adult testicular volume [11]. The earliest stage of maturation is an increase in testicular volume (more than 3 ml), followed by thinning of the scrotum, penile growth, pubic hair development, and, lastly, a linear growth spurt [9]. Pathological changes may be present when pubertal changes have started but fail to get completed within approximately four years of its onset, a condition considered stalled puberty [11]. Hypogonadism is classified as either primary or secondary, primary hypogonadism, also known as hypergonadotropic hypogonadism is caused by testicular failure and is associated with elevated gonadotropin levels. Secondary hypogonadism, also known as hypogonadotropic hypogonadism (HH), is characterized by low or inappropriately normal gonadotropin levels, as well as low testosterone concentrations, and is caused by a hypothalamic or pituitary defect or damage [12]. HH can be transient due to an underlying medical condition or persistent due to a congenital, acquired pituitary disorder or idiopathic in origin [7,12]. Constitutional delay of growth and puberty (CDGP) is the most common cause and is a nonpathological condition where the affected subjects achieve complete sexual maturation later than their peer. There is often a strong family history of CDGP in the parents or siblings, which suggests that there may be an underlying genetic cause [13,14]. 

When and how to evaluate the patient with delayed puberty? 

The initial evaluation aims to rule out underlying disorders causing delayed puberty. Table 1 summarizes the commonest causes of delayed puberty [1,14-16].

**Table 1:** Causes of delayed puberty.

Constitutional Delayed Growth and Puberty (CDGP) 60%-65%
Gonadotropin deficiency (hypogonadotropic hypogonadism) 10%
Isolated gonadotropin deficiency
Idiopathic
Kallmann syndrome (with anosmia)
Genetic (e.g., GNRHR, GNRH1, GPR54, FGFR1, FGF8, PROK2, PROK)
Obesity syndromes: (LEP, LEPR, and PCSK1 mutations), Prader-Willi
Syndrome, and Bardet-Biedl syndrome.
Multiple pituitary hormone deficiencies
Congenital (Commonest Prop1 gene mutation)
Acquired due to central nervous system lesion (e.g., Craniopharyngioma)
Primary gonadal failure (hypergonadotropic hypogonadism) 5%-10%
Radiation to the testes
Following surgery for cryptorchidism
Vanishing testes syndrome
Klinefelter syndrome (small testes but adequate androgen production)
Functional hypogonadotropic hypogonadism 20%
Inflammatory bowel disease
Anorexia nervosa
Celiac disease
Cystic fibrosis
Thalassemia and sickle cell disease
Juvenile rheumatoid arthritis (JRA)
Hypothyroidism
Excessive exercise

Clinical history 

Questions about the initiation and evolution of body odor, acne, testicular growth, and pubic and axillary hair should be asked of patients and their parents. Also, it is important to inquire about the psychosocial impact and emotional stress affecting the patient. A family history should be retrieved, including childhood growth patterns and the parents' age at pubertal onset. It has been estimated that 80% of patients with CDGP have first-degree family members with delayed puberty [17,18]. Underlying secondary disorders can cause temporary delay of puberty (functional HH) if they are of sufficient intensity and duration. Therefore, it is essential to inquire about chronic disease symptoms, with a focus on certain disorders (e.g., poorly controlled type 1 diabetes, celiac disease, severe asthma, thyroid disease, Thalassemia, sickle cell disease, and anorexia) as well as medication use, nutritional status, and psychosocial functioning. Bilateral cryptorchidism or small penis at birth may suggest HH [19]. Also, hyposmia or anosmia may suggest Kallmann syndrome. History of chemotherapy or radiotherapy may indicate primary gonadal failure or HH. Hypogonadism in pediatric cancer patients is linked to the patient's age, treatment dose, and duration. Hypogonadism affects between 11% and 56% of juvenile cancer survivors, according to current estimates [20-22].

Physical examination 

Tanner scale, growth chart, and orchidometer are the tools needed to document and track the development of secondary sexual characteristics and puberty. Generally looking for any dysmorphic features, midline defects, along with obtaining height and weight and plotting the measurements for comparing it with previous ones to assess longitudinal growth is the main part of the examination [1,15,16,23]. The Prader orchidometer is widely used in clinical settings to estimate the testicular volume and is inexpensive, usually correlates well with ultrasonography for testicular size and volume [24]. The clinical findings associated with delayed puberty are summarized in Tables 2, 3. 

**Table 2:** Genitalia's findings associated with delayed puberty.

Genitalia
Testes <2.5 cm in length (volume < 4 mL) are prepubertal.
Penis <7 cm stretched is prepubertal
Penis <5 cm is small and may suggest congenital hypogonadotropic hypogonadism [18].
Bilateral cryptorchidism may suggest congenital hypogonadotropic hypogonadism [18,22].
Pubarche may or may not be present and does not impact a diagnosis of delayed puberty.

**Table 3:** Growth and body proportion findings associated with delayed puberty.

Growth and body proportions
Most boys who have CDGP are <10th percentile in height.
A linear growth curve that is below but parallels to the third percentile, with a drop off after the age of 13 years, is suggestive of CDGP.
Growth rate < 3 cm/year during adolescence may suggest hypogonadotropic hypogonadism, hypopituitarism, growth hormone deficiency, or hypothyroidism but can also occur with CDGP.
Normal weight or being overweight for height is suggestive of CDGP.
Morbid early childhood obesity with normal development suggests leptin pathway gene mutation (LEP, LEPR, and PCSK1 mutations), if delayed development consider Prader-Willi syndrome or Bardet-Biedl syndrome.
Low weight for height is common in boys with an underlying disorder that causes a delay in puberty.
Boys with delayed puberty due to Klinefelter syndrome are usually tall [25].
Boys with persistent hypogonadotropic hypogonadism may have eunuchoid body proportions characterized by arm span greater than height due to late epiphyseal closure [25].

Investigations 

**Figure 2:**
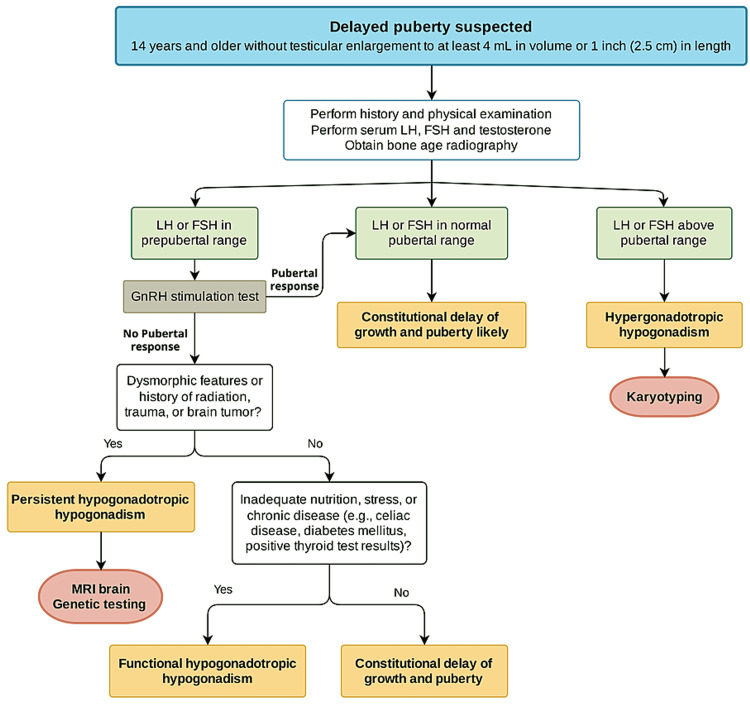
Diagnostic approach to a boy with suspected delayed puberty.

Initial screening tests to confirm the diagnosis and to distinguish between primary and secondary hypogonadism include serum LH, FSH, and testosterone. Thyroid function tests, prolactin, and insulin-like growth factor (IGF-1) are often needed to exclude any underlying disorders that have an impact on the onset of puberty and can delay it. If height velocity does not rise on testosterone therapy or short stature is a feature at the presentation, a diagnosis of growth hormone deficiency must be ruled out. Other labs include complete blood count, erythrocyte sedimentation rate, blood urea nitrogen, creatinine, tissue transglutaminase-immunoglobulin A antibodies (tTG-IgA), and liver function tests should be done to evaluate for the possibility of nutritional disorders, celiac disease, or occult chronic illnesses. A radiograph of the left hand and wrist to evaluate bone age should be obtained at the initial visit to assess skeletal maturation and then repeated over time if needed. Testicular ultrasonography can be used to determine testicular volume, omitting the contribution of the epididymis and overlying skin and providing a more precise estimate, particularly for smaller testicular volumes [24]. Additional tests have been proposed to help in distinguishing between CDGP and congenital HH (CCH) which include inhibin B, antimullerian hormone, basal gonadotropin (LH and FSH) levels, GnRH stimulation, or GnRH-agonist stimulation tests, and human chorionic gonadotropin stimulation tests [26,27]. Depending upon the clinical presentations MRI brain to rule out intracranial tumors or genetic testing may be indicated. In this review we seek to discuss puberty induction in boys, however, a detailed review of diagnostic workup is beyond the scope of this review, and the main diagnostic approach is illustrated in Figure 2.

Puberty induction 

Goals

The current practice of hormonal therapy to induce puberty in adolescent males is based on published consensus and expert opinion. Evidence-based guidelines on optimal timing and regimen in puberty induction in males are lacking, and this reflects some discrepancy in practice among endocrinologists which was reflected in surveys done in 2004 and 2020 [28,29]. Delayed puberty can result in significant psychological distress as well as low self-esteem for the adolescent male [30-32]. Additionally, it has a negative impact not only on metabolic profile, fat distribution, muscle mass, and bone mass but also on growth velocity [33,34]. Sex steroids are vital therapeutically in attaining mid-parenteral height and play an important role in numerous aspects of growth regulation [33,34]. Boys with CDGP typically tend to have short stature and delayed bone maturation. Multiple observational studies including 97 boys with self-limited delayed puberty found near-adult heights that are comparable to expected adult heights or mid-parental heights [35-37]. On the other hand, other studies with a total of 218 boys, suggest that these boys may not reach their genetic height potential [38-40]. Several studies have evaluated the effects of testosterone therapy and reported that it does not adversely affect adult height and that there is no significant difference in adult height between treated and untreated boys [41-43]. Boys with HH who are treated later in life have aberrant body proportions and are taller than average [44]. The principal motivation for treating adolescents with CDGP is the severe psychological suffering they experience as a result of being shorter and less physically developed than their peers. The purpose of managing puberty in CDGP is to improve well-being and optimize growth and final height. In the case of persistent HH maintenance, hormonal replacement therapy is required after puberty induction to prevent adverse effects of delayed puberty on body proportions, to improve peak bone mass, and to avoid metabolic and psychosocial adverse effects associated with delayed puberty and hypogonadism. 

Optimal Timing

Adolescents with pubertal delay should begin puberty induction therapy around the age of normal average puberty (12 years), but boys with CDGP might present later and begin treatment closer to 14 or 15 years of age [45]. Nonetheless, some endocrinologists tend to wait until the patient's bone age is at least 10.5 years old because they are concerned about adult height implications if treatment is started too early [45].

Treatment options for adolescent boys with delayed puberty

To induce puberty, testosterone injections are the most widely used therapy in adolescent males with CDGP or hypogonadism. When compared to other regimens, testosterone is an effective, practical, safe, well-tolerated, and low-cost option. However, the effect of testosterone on the initiation of spermatogenesis and testicular growth is an unresolved issue. However, in adolescent males with hypogonadism, hCG with or without FSH appears to be more physiologic and potentially safer than testosterone in initiating spermatogenesis and testicular growth. In this review, various treatment options will be briefly explored to improve the management of this condition.

Testosterone

In most cases, testosterone is used to induce puberty in boys with hypogonadism and CDGP due to the flexibility in dose administration. A lower dose of testosterone is usually required at first to induce puberty in patients with hypogonadism and CDGP. For those who require long-term treatment, the dose is gradually increased [46,47].

In boys with permanent hypogonadism, testosterone therapy should be started at an appropriate age usually around the chronological age of 12 for physiological induction of puberty [46], while it is around 14 years for patients of CDGP [48]. For patients with concomitant severe short stature, growth hormone deficiency, and delayed bone age, testosterone therapy is usually delayed to allow increasing the final adult height [48]. Most of the clinical data for use in the management of pubertal development has been with the testosterone esters such as T enanthate and cypionate or with a mixture of very short and short-acting esters [46]. There is a paucity of published clinical data in respect to other testosterone formulations in adolescent populations (Table 4). The long-acting intramuscular preparation of T undecanoate for puberty induction and maturation is usually indicated for boys with permanent hypogonadism [49] and is unsuitable for cases with CDGP. A randomized cross-over study of oral vs. intramuscular testosterone did not show any significant difference in terms of efficacy for linear growth between the two agents [50]. Lawaetz et al. showed oral T undecanoate formulations were found to be effective in promoting height, inducing secondary sexual characteristics but without affecting bone age advancement [51]. Moreover, a three-month therapy resulted in a significant increase in fat-free mass along with increased height velocity [52]. Transdermal testosterone was found to be effective in promoting growth and virilization in patients with secondary hypogonadism affected by beta-thalassemia [53]. Similarly, 1% testosterone gel was effective in promoting secondary sexual characteristics in boys affected with Klinefelter’s syndrome [54]. Recently, testosterone transdermal gel preparations in strengths of 1% and 2% were found to be safe and effective on adolescent hypogonadal boys with concomitant hypertransaminasemia [55]. Another recent study on boys affected with CDGP has reported equal efficacy of testosterone transdermal gel 2% and intramuscular testosterone in comparison to untreated subjects in increasing height velocity [56]. For patients with partial androgen insensitivity syndrome (PAIS) and 5-alfa reductase deficiency, Dihydrotestosterone gel 2.5% has resulted in increased penile length [57,58]. Furthermore, a randomized, open-label trial on boys with CDGP compared efficacy of intramuscular testosterone (1 mg/kg/4 weeks) to oral letrozole (2.5 mg/day), for promoting puberty, reported a greater rise in gonadotrophins and testicular growth with letrozole although linear growth and bone age advancement did not differ [59]. The testosterone treatment options for the induction of puberty in boys with CDGP and hypogonadism are presented in Table 4.

**Table 4:** Summary of the studies done in puberty induction.

Testosterone Preparation	CDGP	Hypogonadism
T. Enanthate, Cypionate or a mixture of T. esters, IM injections	Starting dose: 50 mg monthly, for 3-6 months [46,60]	Starting dose: 25-50mg monthly. Increase by 50 mg every 6-12 months [46,60]
	May increase the dosage by 25-50 mg. Maximum Dose 100 mg [46,60]	Adult dosage: 150-200 mg every 2weeks [61]
T. Undecanoate IM Injection	No data available	For puberty induction, only in young men [62]
		Adult dosage: 750-1000 mg every 10-14weeks [61,62]
T. Transdermal gels	10 mg daily for 3 months [56]	Gel 1%: 0.5 g/day, increased up to 5 g/day as needed [54]. Adult dosage: 5-10 g/day [61]
		Gel 2%: Initial dose 10 mg/day [55]. Adult dosage: 40-70 mg/day [61]
T. Undecanoate Oral tablets	Initial dose 40 mg daily, Maximum dose 80 mg twice daily [51]	Adolescent Population: No data
	40 mg daily for 4 weeks [50]	
	40 mg daily for 3 months [52]	Adult with hypogonadism , maximum dose is 80 mg twice daily [51]
	20 mg daily for 6 months [63]	
	40 mg daily for mean of 3.5 months [64]	
T. Transdermal patches	Age 12.5 to 15 years: 5 mg over 8-12 hours for 8 weeks [65]	Pre-pubertal 14-16 years: 2.5 mg over 12 hours overnight [65]
		Partially virilized 17-19 years: 2.5 mg daily [65]
		Virilized men above 20 years: 5 mg daily [53]
T. Pellets Subcutaneous	No Data Available	13.9 to 17.5 years: 8-10 mg/Kg every 6 months for three doses [53]
T. Nasal gel	No Data Available	No Data Available for the adolescent population
T. Transbuccal Bio-adhesive tablet	No Data Available	No Data Available for the adolescent population

Monitoring of Testosterone Therapy in Boys

Testosterone therapy should be increased gradually to mimic normal pubertal physiology and can be stopped once the HPG axis has been significantly activated, as indicated by an increase in the testicular volume of 6 to 8 mL. Adolescents with permanent hypogonadism, on the other hand, require gradual increases in testosterone dose over two to three years until adult doses are reached to allow for optimal growth [45]. For evaluation of the effectiveness of testosterone therapy in clinical practice, regular follow-up every three to six months is needed along with an assessment of progression of pubertal maturation, height velocity, and changes in body composition [46]. Along with clinical assessment, other imaging, and laboratory workups such as bone mineral density assessment by DXA and hand-wrist radiograph for bone age are useful monitoring tools for both therapeutic benefits and side effects of testosterone therapy. Monitoring is highly recommended and has been standardized for testosterone therapy in hypogonadal men in the recent guideline [61], but such clear guidance is lacking for adolescents [46,66] and studies have shown that these adolescents on testosterone therapy undergo insufficient and incomplete biochemical monitoring [67]. Such wide variations in monitoring can be explained based on many diverse conditions and clinical indications in adolescents necessitating testosterone therapy. It is to be acknowledged that guidelines for clinical and hormonal monitoring for patients undergoing testosterone therapy and targeted approaches based on the etiology of hypogonadism are lacking due to the paucity of studies. With the availability of various newer testosterone formulations and the increasing knowledge of its therapeutic effects, careful monitoring and structured guidelines are needed more than ever. In this regard, Stancampiano et al. proposed a practical approach in a recent article [68]. Based on the temporary or permanent need for ongoing testosterone therapy, they proposed two different schemes for monitoring of replacement therapy and recommended complete blood count, liver function tests, bone age assessment with full clinical evaluation before starting testosterone therapy. This will provide us with not only a baseline, but also the ability to identify underlying diseases such as polycythemia or hypertransaminasemia when testosterone therapy is contraindicated. It will also alert the physician to additional diagnostic work-up for an underlying condition and the selection of preparations with a lower side effect profile, paving the way for individualized monitoring for each patient. Boys with CDGP usually have induction of puberty with a six-month course of testosterone therapy and once the therapy is initiated, full clinical evaluation alone at three- and six-month intervals is usually sufficient. Patients with a strong suspicion of CDGP but who are requiring therapy for more than six months need to be managed differently. They will need thorough evaluation for the underlying etiology and are likely to do better with the monitoring protocol for patients with hypogonadism which includes assessment of bone mineral density along with the metabolic and gonadal profile. Monitoring in cases with permanent hypogonadism requires the bone mineral density assessment by DXA scan using validated methods with adjustment for age, size, and sex along with bone age assessment and serum lipid profile at baseline, one year, and then every one to two years. Thorough clinical evaluation and laboratory assessment such as serum total testosterone and complete blood counts needs to be done at baseline and at three, six, and 12 months followed by periodic assessment every 6-12 months has been suggested while liver function tests and serum level of FSH and LH is to be obtained at baseline [67-69]. The psychosocial impact can be assessed with a standardized QoL tool such as the EQ-5D-Y [70]. The assessment of the effectiveness of testosterone therapy in clinical practice should be based mainly on the clinical response observed such as tanner stage progression (increase in stretched penile length) and development of secondary sexual characteristics (deepening of the voice, muscle mass accretion, facial and body hair growth). However, biochemically, keeping serum total testosterone level in the mid-normal reference range during treatment is much safer for the pubertal stage [46] forming the basis of the recommendation for its laboratory assessment which needs to be done periodically as mentioned above. For testosterone therapy, depending upon the type of preparation used and the timing of its administration, variability is observed in the level of serum testosterone obtained. For intramuscular testosterone enanthate or cypionate, the sample should be collected between the injections, while for testosterone undecanoate, it should be collected before the next dose. The level of testosterone is usually checked two weeks after starting therapy and 3-12 hours after application of transdermal testosterone patch while for transdermal gel preparations, it should be tested two hours after application, two weeks after starting treatment. In case of oral testosterone undecanoate, the level can be checked in a non-fasting state 3-5 hours after ingestion and after at least two weeks of starting therapy.

Potential adverse effects of testosterone replacement

Adverse effects of testosterone therapy are uncommon in the short-term therapy of three to six months usually indicated for induction of puberty; however, they can occur in those with hypogonadism where long-term therapy is indicated. Erythrocytosis, acne and oily skin, Detection of subclinical prostate cancer, growth of metastatic prostate cancer, and reduced sperm production and fertility are some of the effects for which there is evidence of association with testosterone therapy, while gynecomastia, male pattern balding, growth of breast cancer and induction or worsening of obstructive sleep apnea are few of the uncommon adverse events having weak evidence of association with testosterone therapy [61].

Gonadotropin

The stimulation of testicular growth and spermatogenesis with improvement in potential fertility is an additional benefit of gonadotropin treatment over testosterone treatment [71]. Although it is commonly used to treat infertility in adults with CHH, it can also be used to induce puberty in adolescent males with CHH. On the other hand, for an inpatient with CHH, testosterone therapy alone is not a feasible treatment option for stimulating testicular growth. 

To induce puberty in adolescent boys with CHH, various treatment protocols have been used, including hCG alone or in combination with FSH. The treatment regimen varies between 1,000-1,500 IU for hCG and 75-150 IU for FSH administered intramuscularly three times per week [72]. The hCG dose is to be titrated based on testosterone levels, whereas the FSH dose is usually adjusted based on clinical signs [72]. In a retrospective study of boys with CHH, treatment with 5,000 in weekly hCG injections and monthly testosterone injections had a comparable virilizing effect but the final testicular volume was significantly greater in patients treated with hCG [73]. Nonetheless, a prospective study including adolescents with delayed puberty, the majority of them with absent puberty, the use of hCG and rFSH led to significant testicular growth and induced spermatogenesis in 91% of patients [74].

Using FSH alone may be considered in adolescents with severe GnRH deficiency where the goal of priming with FSH alone is to stimulate the proliferation of immature Sertoli cells before seminiferous tubule maturation [75,76]. Raivio et al. [77] studied a small group of boys aged 9.9-17.7 years with gonadotropin deficiency who were initially treated with FSH alone (two mo-2.8 years) that induced testis growth and increased circulating inhibin B levels, followed by successful pubertal induction with a combination of hCG and r-hFSH. Furthermore, a randomized controlled study of 18 adolescents GnRH-deficient men (CHH) with prepubertal testes (<4 mL) and no cryptorchidism or prior gonadotropin therapy showed FSH pre-treatment followed by GnRH was successful in inducing testicular growth, normalizing inhibin B levels, and promoting fertility [78].

Pre-treatment with FSH prior to testicular maturation appears to compensate for suboptimal testicular development during late fetal life and mini puberty, and thus may be beneficial for optimizing testicular growth and future fertility in adolescent males. It was previously noted that the initial testis size in men with CHH reflects the degree of gonadotropin deficiency and predicts treatment response [79]. Thus, boys with complete gonadotropin deficiency as determined by initial mean testicular volume < 4 mL require both hCG and FSH to achieve full testicular maturation, whereas boys with partial gonadotropin deficiency with initial mean testicular volume, 7 mL usually require only hCG [80].

Gonadotropin-releasing hormone

Throughout puberty, the LH and FSH response increases with the progression of puberty, GnRH stimulates the release of both LH and FSH [81]. Pulsatile GnRH treatment may be an option for patients with CHH who have GnRH deficiency but normal pituitary function. The most physiological approach is to use GnRH infused in a pulsatile fashion, with pulse intervals of 90-120 minutes. I.V. infusion results in the most effective pulsatile stimulation and thus the pulsatile release of gonadotropin, whereas sc administration results in more flattened gonadotropin levels, which can also result in adequate gonadal stimulation [82]. For hypogonadotropic males, GnRH treatment will result in a complete development with testicular growth including spermatogenesis and virilization [83]. For optimal testicular growth and spermatogenesis, the individual dose of GnRH and the time required to achieve maximum effectiveness are variable, ranging from 25-600 ng/kg and requiring a minimum of two years [84]. According to Liu et al. [80], pulsatile sc GnRH therapy for two years in adolescents with the complete form of CHH does not significantly accelerate or enhance testicular growth, hasten the onset of sperm production, or increase sperm output compared to hCG/hMG therapy. Thus, whether pulsatile GnRH administration, a more time-consuming treatment modality, does not offer any practical advantages over conventional hCG/hMG therapy in men with idiopathic HH (IHH), especially given the latter's extremely high fertility rate, remains to be seen [85].

Our suggested approach to patient delayed puberty

There has been significant variation in the induction of puberty of adolescent males with central hypogonadism, and there is little agreement on proper treatment. A small number of studies, primarily in those with permanent hypogonadism, support our practice. We used a variety of treatment regimens for pubertal induction and completion, all of which were based on our experience rather than evidence provided by carefully designed studies. After several years of clinical practice, these regimens appear to be largely successful in achieving full virilization.

**Figure 3:**
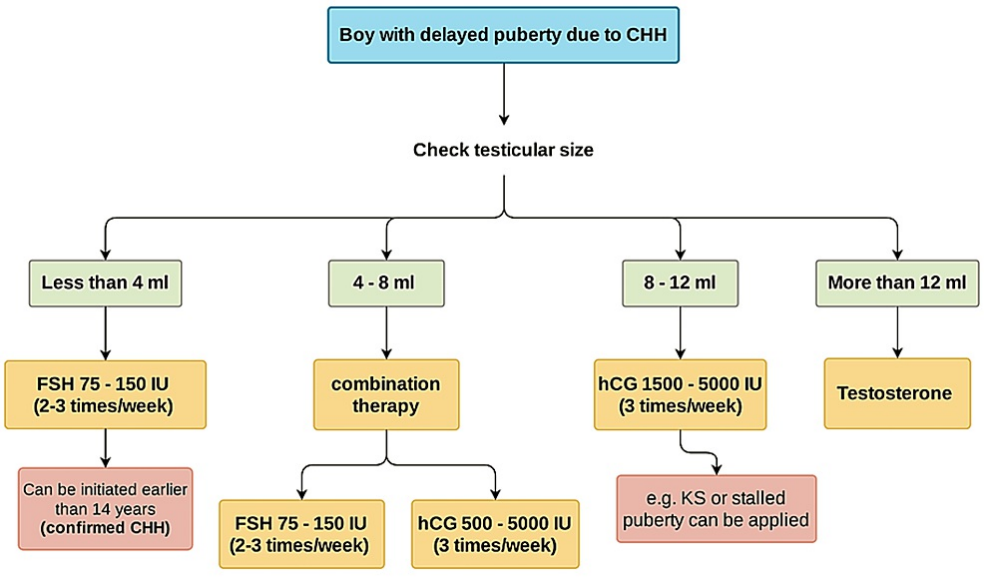
Suggested treatment approach for patients with delayed puberty due to HH (complete or stalled puberty).

The initial testicular size usually reflects the severity of gonadotropin deficiency and predicts the increase in testicular volume in response to treatment in patients with delayed puberty and hypogonadism, so we use it as a guide for selecting the initial treatment option. We use both hCG and FSH in an adolescent boy with a previous history of absent puberty and small testicular size. Although hCG alone can increase testicular volume, combined treatment with hCG and FSH have been shown to result in a better response in terms of final testicular size, because normal levels of both gonadotropins appear to be necessary for appropriate spermatogenesis induction during puberty. If the patient's pubertal development occurs spontaneously and the testicular size is greater than 4 mL, hCG can be started as a monotherapy. When a patient lacks pubertal development and has a testicular size of fewer than 4 mL, the optimal treatment regimen to optimize testicular growth and maximize the potentiality for fertility is unknown; however, we usually begin with FSH as monotherapy; hCG can be added if the patient achieves better testicular growth. Subsequently, we switch to testosterone in both groups when the testicular volume reaches the normal adult range or no further increase in testicular size was obtained. Figure 3 summarized the treatment approach for patients with delayed puberty due to HH (complete or stalled puberty).

**Figure 4:**
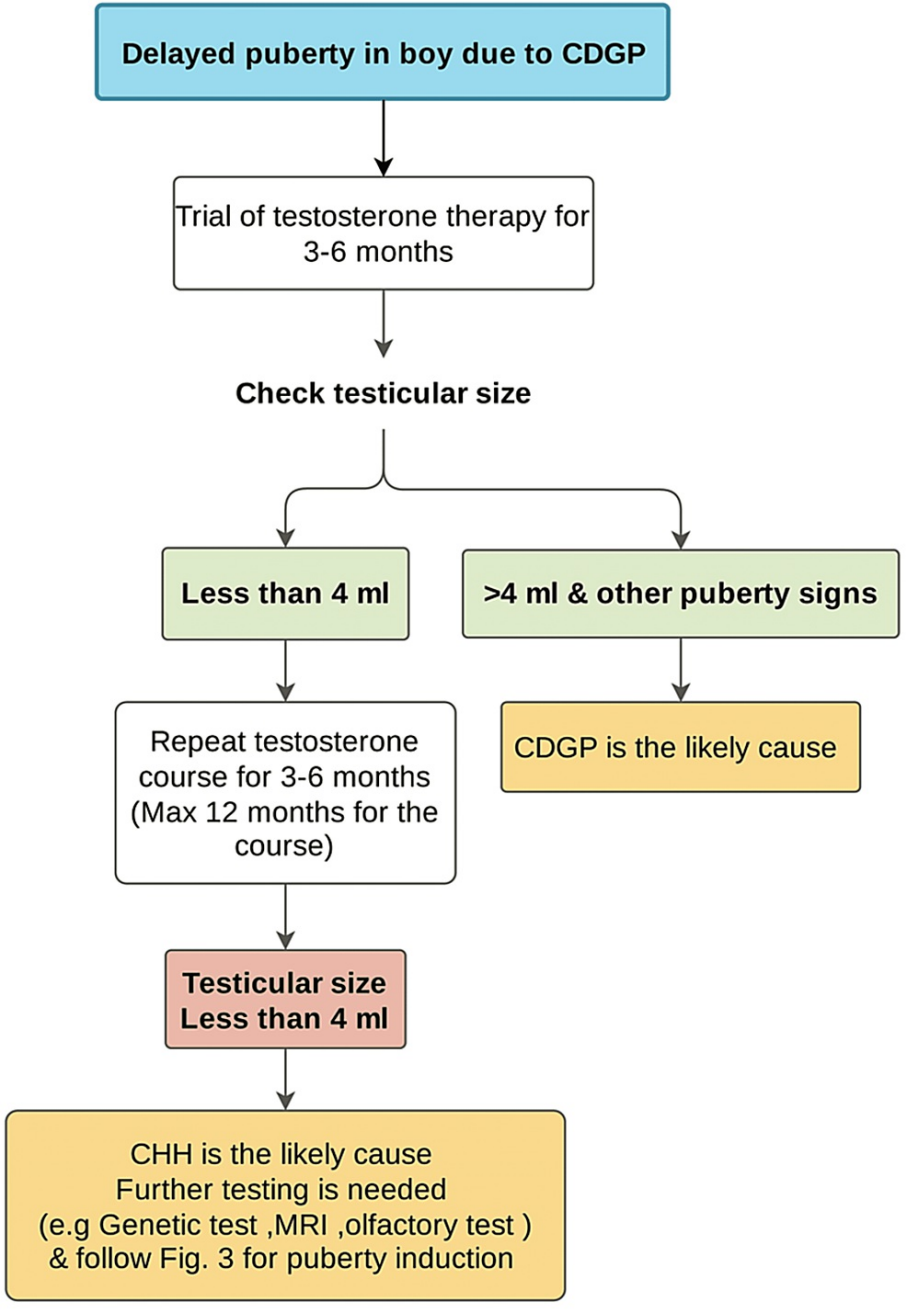
Suggested treatment approach for patients with delayed puberty due to CDGP. CDGP - Constitutional delay of growth and puberty

**Figure 5:**
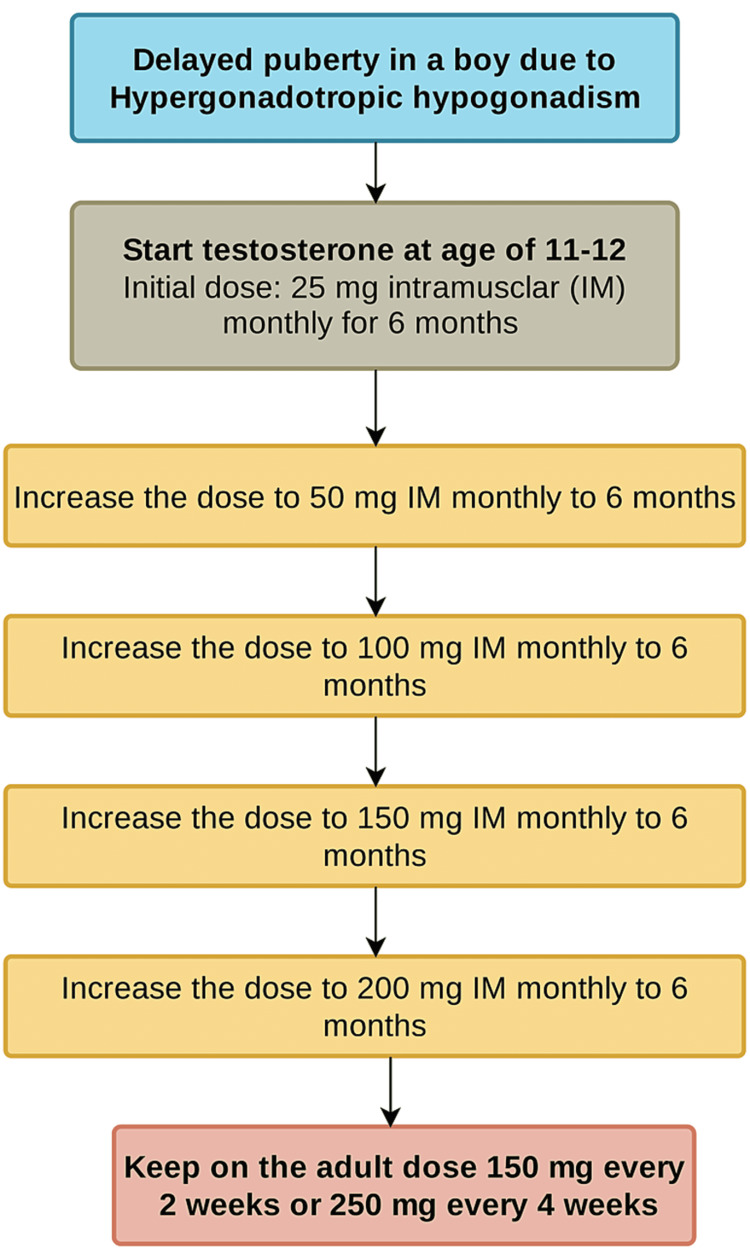
Hormonal replacement in hypergonadotropic hypogonadism (low testosterone, high LH and FSH) (e.g., Klinefelter syndrome 47 XXY mixed gonadal dysgenesis 46 X0, XX, congenital anorchia and testicular postradiotherapy).

However, the treatment is usually easier for older boys with delayed puberty and adult testicular size. Expectant observation or low-dose testosterone therapy are the two treatment options for CDGP patients. Figure 4 summarized the treatment approach for patients with delayed puberty due to CDGP. For patients with delayed puberty due to hypergonadotropic hypogonadism, our approach is to start testosterone at the age of 11-12 years old and gradually increase. Figure 5 summarized the treatment approach for patients with delayed puberty due to hypergonadotropic hypogonadism.

## Conclusions

Adolescence is a critical period in human life, marking the transition from childhood to emerging adulthood and characterized by numerous challenges and developments in both the physical and social domains. Testosterone therapy in adolescent boys is primarily intended to increase linear growth and pubertal progression, but it may also improve bone mineral content, muscle function, metabolic profile, and psychological well-being. Some people may only need testosterone therapy for a short time, while others may need it for the rest of their lives, and therapy monitoring will thus depend on the underlying condition. Gonadotropin treatment can also be used to induce puberty in an adolescent male with hypogonadism. The stimulation of testicular growth and spermatogenesis with improvement in potential fertility is an additional benefit of gonadotropin treatment over testosterone treatment.
